# Review of factors resulting in systemic biases in the screening, assessment, and treatment of individuals at clinical high-risk for psychosis in the United States

**DOI:** 10.3389/fpsyt.2023.1117022

**Published:** 2023-03-13

**Authors:** Miranda A. Bridgwater, Emily Petti, Maksim Giljen, LeeAnn Akouri-Shan, Joseph S. DeLuca, Pamela Rakhshan Rouhakhtar, Caroline Millar, Nicole R. Karcher, Elizabeth A. Martin, Jordan DeVylder, Deidre Anglin, Raquel Williams, Lauren M. Ellman, Vijay A. Mittal, Jason Schiffman

**Affiliations:** ^1^Department of Psychological Science, University of California, Irvine, Irvine, CA, United States; ^2^Department of Psychology, University of Maryland, Baltimore County, Baltimore, MD, United States; ^3^Department of Psychological and Brain Sciences, Fairfield University, Fairfield, CT, United States; ^4^Department of Psychiatry, Washington University School of Medicine, St. Louis, MO, United States; ^5^Graduate School of Social Service, Fordham University, New York, NY, United States; ^6^Department of Psychology, The City College of New York, New York, NY, United States; ^7^Thrive Together OC, Orange, CA, United States; ^8^Department of Psychology and Neuroscience, Temple University, Philadelphia, PA, United States; ^9^Department of Psychology, Northwestern University, Evanston, IL, United States

**Keywords:** social determinants, clinical high-risk, psychosis, screening, systemic bias

## Abstract

**Background:**

Since its inception, research in the clinical high-risk (CHR) phase of psychosis has included identifying and exploring the impact of relevant socio-demographic factors. Employing a narrative review approach and highlighting work from the United States, sociocultural and contextual factors potentially affecting the screening, assessment, and service utilization of youth at CHR were reviewed from the current literature.

**Results:**

Existing literature suggests that contextual factors impact the predictive performance of widely used psychosis-risk screening tools and may introduce systemic bias and challenges to differential diagnosis in clinical assessment. Factors reviewed include racialized identity, discrimination, neighborhood context, trauma, immigration status, gender identity, sexual orientation, and age. Furthermore, racialized identity and traumatic experiences appear related to symptom severity and service utilization among this population.

**Conclusions:**

Collectively, a growing body of research from the United States and beyond suggests that considering context in psychosis-risk assessment can provide a more accurate appraisal of the nature of risk for psychosis, render more accurate results improving the field's prediction of conversion to psychosis, and enhance our understanding of psychosis-risk trajectories. More work is needed in the U.S. and across the globe to uncover how structural racism and systemic biases impact screening, assessment, treatment, and clinical and functional outcomes for those at CHR.

## 1. Introduction

Psychosis-spectrum disorders represent a severe and debilitating type of mental health concern, with prevalence estimates ranging from 0.25 to 0.64% in the United States (1). These disorders are conceptualized as existing on a severity spectrum on which subthreshold, psychotic-like experiences and symptoms (often lacking the distress, full conviction, or functional impairment required to meet diagnostic criteria for psychosis) are placed on one end, while full-threshold, diagnosable psychotic disorders are placed on the other (2, 3). Individuals, typically adolescents and young adults, reporting distressing or impairing subclinical psychotic symptoms are sometimes referred to as being at clinical high risk (CHR) for psychosis. The CHR phase is characterized by attenuated positive psychotic symptoms that do not occur at levels of severity or frequency that constitute a full threshold psychosis-spectrum disorder (e.g., unusual thought content or perceptual abnormalities that are attenuated forms of delusions or hallucinations) (4). In the United States, CHR status is typically assessed by the Structured Interview of Psychosis-Risk Syndromes (SIPS) (4, 5), the gold-standard, clinician-led interview of psychosis-risk symptoms. Recent literature has suggested that specialized early interventions, particularly when applied in the CHR phase, may lessen the later severity or even prevent the onset of full-threshold psychotic disorders (6–8). With a focus on work emanating from the United States, this paper presents a narrative review of sociocultural factors thought to impact symptoms, screening, assessment, and treatment of individuals at CHR for psychosis. We speculate that factors reviewed below may pose challenges for clinicians regarding implicit bias in CHR assessment and differential diagnosis. We offer a range of suggestions to minimize the impact of these possible challenges.

### 1.1. Narrative review approach

In response to a call for manuscripts, “Advances in Identifying Individuals at Clinical High Risk (CHR) for Psychosis: Perspectives from North America,” we constructed a narrative overview of sociocultural factors thought to impact risk for psychosis in the U.S. and Canada. Though research highlighting these factors as they impact populations at risk living in North America has been increasing, most of the relevant literature focuses on samples recruited within the United States. Due to this, most of the research cited originates from the United States. To best meet the goals of this manuscript, we pursued a narrative review approach, drawing upon other recent reviews, published original work, calls to action, and commentaries [see (9–12)] to identify several specific sociocultural factors that may be most relevant and particularly salient for North American and United States populations. We then screened and selected articles from the reference lists of these papers. We also conducted literature searches on selected topics to identify additional studies related to these factors. Where CHR-specific literature was limited or unavailable, we cite research on psychotic-like experiences (PLEs), subclinical forms of psychosis-spectrum experiences that have not been assessed through clinician-led CHR interviews. After reviewing the literature, we present the ways in which these factors may lead to implicit bias in CHR screening and assessment, potentially resulting in incorrect labeling of normative experiences as CHR. Where applicable, we also present issues of differential diagnosis. As implicit biases may impact differential diagnosis and vice versa, we highlight where these two barriers to accurate assessment and treatment may be intertwined and difficult to disentangle. These discussions are followed by “Clinical Considerations”, in which concrete suggestions and tools to incorporate these factors into assessment and clinical practice are provided (see Table 1).

**Table 1:** Clinical considerations for each contextual factor reviewed.

**Contextual factor**	**Clinical considerations**
Racialized identity and ethnicity	• Incorporate inclusivity initiatives in CHR clinics to make services more accessible to racial/ethnic groups less likely to seek or receive care (e.g., provide information in multiple languages, hire staff from diverse backgrounds, host regular diversity continuing education workshops) • Improve accuracy of screening and assessment tools across racial/ethnic groups • Employ interviews or follow-up questions when using screens with weak predictive validity among the client's racial/ethnic group • Consider whether endorsement of CHR experiences meets the essence of the CHR construct or is better explained by context • Probe whether experiences endorsed are common among the client's racial/ethnic group, community, and family • Use tools such as the Cultural Formulation Interview (13) to gather relevant cultural and contextual information • Follow-up questions with client could include: ∘ “You said ‘yes' to my question about X. Tell me why you said yes to that one?” ∘ “How does your experience compare to the experiences of others from similar backgrounds as you?”
Discrimination	• Query for experiences of discrimination alongside CHR through interview or self-report [e.g., Perceived Discrimination Scale (14); Multiple Discrimination Scale (15); Experiences of Discrimination measure (16)] • Ask clients directly and sensitively about discrimination they may have experienced • Incorporate an understanding of client's experiences with discrimination when forming conceptualizations of symptoms and subsequent treatment recommendations • Consider whether psychosis-risk endorsements (e.g., feelings of being targeted or paranoia-like symptoms) may be normative reactions to having experienced discrimination • Be sensitive to the possible role of intergenerational discrimination and its possible impact on your client • Remain mindful that normative responses to discrimination can be mistaken for CHR symptoms leading to overdiagnosis AND discrimination can lead to stress that can contribute to CHR, running a risk of underdiagnosis • Follow-up questions with client could include: ∘ “Have you or others close to you experienced discrimination or unfair treatment? In what settings or situations?” ∘ “Does that experience only happen when people treat you or others like you unfairly?”
Neighborhood context	• Ask neighborhood-specific follow-up questions during clinician-led interviews to avoid over-pathologization of normative behaviors in response to neighborhood context OR under-pathologization based on assumptions about one's neighborhood context • Incorporate knowledge of local neighborhoods in clinician onboarding, including crime rates, demographic make-up, and geographics • Follow-up questions with client could include: ∘ “Do others in your neighborhood or community feel similarly about this experience?” ∘ “Do you feel you worry about this more than others in your neighborhood or community?”
Trauma	• Be equipped to provide trauma-informed care to those at CHR • Incorporate awareness into CHR assessment that the sequelae of trauma may be mistaken for CHR symptomatology, AND that stress from trauma may lead to the development of true CHR symptoms • Conduct trauma and PTSD assessments alongside CHR assessments to both screen for trauma and disentangle trauma-related and psychosis-risk symptoms, especially for symptoms that may appear in both trauma and psychosis phenomenology (e.g., dissociation) • Be cautious of pathologizing warranted fear or mistrust of law enforcement while remaining sensitive to the distress related to interactions with the police among populations who have experienced disproportionate harm from law enforcement • Assess possible PTSD symptoms in response to exposure to violence by conducting a trauma screener or the trauma module from the Structured Clinical Interview for DSM-5 Disorders (SCID) (17) • Follow-up questions with client could include: ∘ “You said ‘yes' to my question about X. Tell me why you said yes to that one?” ∘ “Does the symptom only occur in objectively dangerous settings or around individuals who have perpetrated violence?” ∘ “Does the symptom occur in any environment?” ∘ “Did the symptom begin before exposure to violence/police victimization, or worsen after exposure to violence/police victimization?” ∘ “How much functional impairment is the symptom causing?”
Immigration status	• Consider migration-related stress during CHR assessment • Ask probing questions about temporality of symptoms about migration • Ask probing questions about whether symptoms endorsed are common or normative to the individual's community and culture of origin • Address the unique barriers to care facing migrants (e.g., access and lack of cultural awareness among providers) • Address provider limitations and biases by holding workshops or seminars to promote cultural responsiveness • Use tools such as the Cultural Formulation Interview (13) to gather relevant cultural and contextual information • Assess for natural supports and community strengths • Follow-up questions with client could include: ∘ “Did the symptom begin before or after you migrated? Did it worsen after immigration?” ∘ “Do others in your culture have this experience?”
Gender identity	• Educate clinicians about types of psychosis-risk experiences that may be more normative experiences in gender-diverse populations (e.g., loss of sense of self; somatic or body-related unusual thought content) • Use probing follow-up questions to differentiate between normative experiences of gender dysphoria and body dysmorphia that may result from societal influence vs. psychosis-related somatic anxiety • Practice gender inclusivity in assessment and treatment settings (e.g., respecting pronouns and preferred names) • Educate clinicians about gender identity and expression and recognize societal issues at a national level that could scare or cause stress for your client locally • Follow-up questions with client could include: ∘ “Have you ever experienced discrimination because of your gender identity? Do you feel this experience is related to that discrimination, or does it feel separate?” ∘ “Have you ever experienced gender dysphoria or body dysmorphia? If so, does the experience you're describing now feel similar to that, or does it feel separate?” ∘ “When did this experience begin? Were other stressors going on in your life around the same time?”
Sexual orientation	• Educate assessors and clinicians about the history of pathologization of queerness and historical maltreatment of sexual minority populations in the health care system • Be transparent about reasons for collecting personal information related to sexual orientation to build trust and empower participants to share their experiences • Consider experiences that may be normative for sexual minority individuals vs. experiences indicative of CHR phenomenology (e.g., developing identity vs. loss of sense of self; unusual vs. excessive guilt related to queer identity) • Avoid making assumptions about sexual orientation (e.g., assuming gender of client partners) • Recognize threat of stress related to homophobia nationally, locally, and within close circles (e.g., family) and how that stress might contribute to risk for psychosis or psychotic-like experiences • Follow-up questions with client could include: ∘ “Have you ever experienced discrimination related to your sexual orientation? Does this experience feel related to that, or is it different?” ∘ “Does the experience you're describing here only occur in situations where you feel you're being targeted because of your sexual orientation, or does it happen outside of those situations too?” ∘ “Do others in your community who identify as queer feel this way as well, or do you feel this way more than they do? Do you feel this way more than those in your community who do not identify as queer?” ∘ “When did this experience begin? Were other stressors going on in your life around the same time?”
Age	• Ask follow-up questions during clinician led interviews to ensure that: ∘ Respondent and interviewer have a shared understanding of the questions ∘ Interview is appropriately differentiating true psychotic experiences (e.g., delusion of mind reading) from developmentally normative experiences (e.g., feeling so close with a best friend that they feel they know what the other is thinking/feeling) • Assess or query for distress surrounding apparent CHR symptoms to differentiate from developmentally normative experiences • Follow-up questions with client could include: ∘ “You said ‘yes' to my question about X. Tell me why you said yes to that one?” ∘ “How does that experience compare to the experience of your friends who are similar in age to you?”

### 1.2. Clinical high risk for psychosis and the role of contextual factors

Approximately 22% of individuals at CHR develop full-threshold psychosis within 3 years of CHR diagnosis (18). With the promise of early intervention strategies, most of which are based on psychosocial approaches to care, it is crucial to understand how context and environment factor into CHR screening, assessment, and services, and where such factors may pose challenges to unbiased assessment and the process of differential diagnosis. This paper will review literature that focuses on these processes in the U.S., occasionally contrasting findings and literature from other parts of the world.

Attempts to understand the impact of sociodemographic, environmental, and contextual factors on the CHR phase have led to several conceptual and theoretical models worldwide that reflect the relevance of such factors in assessment, symptom severity, and mental health care utilization along the psychosis spectrum [e.g., (2, 19, 20)]. For example, the psychosis proneness–persistence–impairment model highlights the role that “environmental risk” plays in the development of more severe, persistent, and clinically relevant psychotic symptomatology (2). Similarly, Petti et al. highlight the role of sociocultural and contextual factors across ecological systems levels in service use for individuals experiencing psychosis-spectrum symptoms (19). Additionally, recent work, such as a review by Anglin et al. (10) of neighborhood factors, trauma, and perinatal factors, has laid important groundwork for examining the links between structural racism, social determinants, and CHR. Due to concerns about the high rates of false-positive results from psychosis-risk screening tools, this review aims not only to synthesize research on the selected contextual factors across the psychosis-spectrum (PLEs, CHR, and psychotic disorders) but also to discuss how these factors may impact psychosis-risk screening and assessment, specifically highlighting the ways these factors may impact screening and assessment in a U.S. context.

## 2. Specific contextual factors

### 2.1. Racialized identity and ethnicity

Most research regarding racialized/ethnic identity and psychosis-spectrum symptoms or diagnoses has been conducted in reference to full-threshold psychotic disorders. A substantial body of research focuses on more frequent or more severe diagnosis of psychosis and schizophrenia in Black Americans compared to White Americans (21–23). While increased exposure to social stress and systemic racism may increase psychotic symptom severity among racially marginalized groups (14, 24), clinician bias and perceptions have also been implicated in differential diagnostic rates (22, 25) such that clinicians are more likely to attribute symptoms to psychosis phenomenology in Black Americans compared to White Americans.

While differential diagnostic rates of CHR by racialized/ethnic identity is a newly developing area of research, there has been a recent increase in research examining racial/ethnic differences in the endorsement of psychotic-like experiences (PLEs), which are conceptually linked to the CHR phase. Karcher et al. (26) found that among a large sample (*N* = 10,839) from the Adolescent Brain Cognitive Development (ABCD) Study of children ages 9–10 years, Black, Hispanic, and multiracial youth reported higher levels of PLEs than Asian and White participants. Experiences of discrimination partially explained this finding. Similarly, DeVylder et al. (27) found elevated levels of PLEs among Black and Hispanic participants compared to White participants and those identifying with other racialized/ethnic groups. These differences were explained by socio-environmental factors, including exposure to discrimination and police violence. Differences in mental health care utilization across racialized groups among individuals reporting high levels of PLEs have also been observed such that college students who self-identified as Asian, Asian American, Black, or African American were significantly less likely to have received past or current mental health care and were considering seeking future services significantly less than those who self-identified as White or European American (19).

Several studies have identified differences in CHR symptom severity (28), recovery rates (29), and social functioning (30) across racialized/ethnic groups. Thompson et al. (28) found more severe positive symptoms among individuals at CHR who identified as racial/ethnic minorities compared to White participants (at trend level). In a study by Salokangas et al. (29), identifying as White was predictive of improved psychosocial outcomes at follow-up visits among participants at CHR. Similarly, Corcoran et al. (30) observed lower levels of social functioning among participants at CHR identifying as racial/ethnic minorities.

Research has suggested that psychosis-risk screening and assessment measures do not perform equally across racialized/ethnic groups, potentially resulting in systematically embedded bias in assessment. For example, Millman et al. (31) found evidence that the PRIME Screen (32), a widely-used psychosis-risk self-report screening measure, predicted interview-based CHR status for White participants but not for Black participants, suggesting that the PRIME Screen may not be an appropriate tool to screen for CHR among Black youth as using the PRIME Screen among Black youth could result in improperly labeling normative experiences as psychosis risk. Similarly, Rakhshan Rouhakhtar et al. (33) assessed the relation of PRIME Screen items with mental wellbeing. They found that only one out of twelve items on the PRIME Screen (“I have been concerned that I might be ‘going crazy”') was negatively associated with mental wellbeing for Black participants, while seven items significantly correlated with mental wellbeing scores among White participants. These findings suggest psychometric differences in a widely used psychosis-risk screening tool across racialized groups that may result in systemic bias in CHR assessment. Overall, there appear to be meaningful differences in CHR screening, assessment, symptom severity, functioning, and recovery across racialized groups. The mechanisms (e.g., social stress, discrimination, and clinician bias) behind these differences, however, remain unclear.

#### 2.1.1. Clinical considerations

The prevalence of over-pathologizing and misdiagnosing psychosis-spectrum disorders among individuals identifying as racial/ethnic minorities combined with evidence that screening measures for CHR differ in their predictive accuracy across racial/ethnic groups strongly suggests that clinicians and researchers alike must take racialized and ethnic identity into account to minimize bias when assessing and treating CHR. It is advisable to consider the predictive validity of screening tools and incorporate additional follow-up questions via interviews about individual item endorsement. Additional work should be conducted to improve screening and assessment accuracy across different racial/ethnic groups. This could involve creating new or refined instruments, or tailoring assessments to various populations.

Considering racial and ethnic background may provide important context for CHR symptoms endorsed during clinical interviews and help avoid bias leading to over-pathologizing (34). For example, certain symptoms, such as feeling suspicious of others or being targeted, may be more attributable to normative suspiciousness in response to experiences of race-based violence in the media or personal experiences of racial/ethnic discrimination (see section 2.2), rather than actual CHR symptomatology. Clinicians and assessors may ask questions to explore whether or not experiences endorsed are shared among the client's racial/ethnic group and use strategies or structured tools [e.g., the Cultural Formulation Interview (13)] to gather relevant contextual information.

Moreover, emerging evidence suggests racial/ethnic differences in mental health care utilization among those experiencing psychosis-spectrum symptoms. Though more research is needed on service use trends within the CHR phase specifically, existing research suggests that it may be beneficial for service providers and clinical settings to promote inclusive practices, such as holding provider trainings focused on reducing ethnocentric biases (35). Additionally, it may be useful for providers to be educated about the impacts of systemic racism on help-seeking, access to care, and service engagement, such as historical abuse of racially marginalized groups within healthcare systems (36). Considering such factors may help to create clinical environments that better serve diverse communities.

### 2.2. Discrimination

Increasing attention has been given to examining discrimination's role in CHR symptom severity and transition to psychosis. Several studies examining discrimination and CHR come from the North American Prodrome Longitudinal Study (NAPLS-2), the largest study of individuals at CHR in North America. In a 2014 manuscript with 540 participants from the NAPLS-2 cohort, those at CHR reported significantly higher levels of perceived discrimination than healthy control participants (37). Findings from a follow-up study of 764 participants at CHR from the NAPLS-2 study (38) also suggested that participants at CHR perceived significantly more discrimination than their healthy control counterparts. Perceived discrimination also appeared to significantly predict transition from CHR to psychosis in the sample, implicating discrimination as a potentially important factor in the developmental trajectory of psychosis-spectrum disorders. These findings suggest that perceived discrimination should be assessed among individuals at CHR, and likely has implications for individualized clinical formulation and comprehensive treatment planning.

Although studies of racial discrimination related to the CHR phase are limited in North America, studies of PLEs can provide insight into the possible relation between discrimination and attenuated forms of psychosis. Over the past decade, evidence has mounted from studies of PLEs in the United States that suggest an association between discriminatory experiences and psychosis-spectrum symptoms. Anglin et al. (39, 40) observed a significant relation between self-reported experiences of racial discrimination and total number of positive symptoms and distressing positive symptoms endorsed on the Prodromal Questionnaire (PQ) among a sample of racial/ethnic minority college students. Among a national survey sample of African American adults in the U.S., several specific domains of racial discrimination—experiencing police abuse, being denied a loan, and being denied a promotion—correlated with endorsing lifetime PLEs (41). This study also identified specific types of discrimination as predictors of PLE subtypes, including visual hallucinations, auditory hallucinations, and delusional ideation. Similar results were also found in a younger sample of 9- and 10-year-olds from the ABCD Study, where greater endorsement of discrimination was associated with higher levels of PLEs (26). In a previously-cited study by Rakhshan Rouhakhtar et al. (33), experiences of discrimination were also significantly associated with certain items on the PRIME Screen for both Black and White participants.

Taken together, studies of PLEs suggest a significant relation between discrimination and psychosis-spectrum symptoms. A handful of studies have suggested that this association holds for individuals meeting criteria for CHR. Research in this area has shifted from examining discrimination as an independent predictor of psychosis-spectrum symptoms to examining discrimination as an explanatory mechanism behind the link between racialized/ethnic identity and psychosis-spectrum symptoms. More research is still needed to better understand the extent to which experiences of discrimination may cause or increase risk for psychosis-spectrum symptoms vs. leading to false positives on psychosis-risk screening tools or interviews.

#### 2.2.1. Clinical considerations

Considering experiences of discrimination can provide important context when assessing and treating CHR symptoms. Discrimination may partially explain the link between differential rates of psychosis-spectrum symptoms across racial/ethnic groups (26). Thus, psychosis-risk assessors and clinicians may benefit from including discrimination measures in CHR symptom conceptualizations. This could be done by assessing experiences of discrimination through brief interviews or self-report forms in tandem with psychosis-risk assessments (see Table 1). In particular, screening and assessment approaches should consider the possibility that normative reactions to discriminatory experiences could be misinterpreted as attenuated symptoms of paranoia or suspiciousness.

On the other hand, in line with stress-vulnerability models of psychosis, experiences of discrimination may contribute to the development of psychosis-spectrum symptoms as a form of social or environmental stress (42). Clinicians and assessors are encouraged to ask clients about discriminatory experiences in a manner that is both sensitive and direct. For example, if a client reports feelings of being targeted or paranoia-like symptoms, assessors and clinicians may ask if the client has faced overt discrimination in the past to probe how these experiences may relate to symptom presentation. This may aid in building rapport, creating a clearer clinical picture, and disentangling discrimination-related experiences from psychosis-spectrum symptomatology.

### 2.3. Neighborhood context

Recent attention has been given to understanding neighborhood contexts and their associations with psychosis-spectrum symptomatology, particularly assessments of suspiciousness and paranoia. As outlined by Anglin et al. (10), neighborhood context encompasses a broad range of variables, including urbanicity, crime, ethnic density, and relative accessibility of resources. In a study of mostly African-American help-seeking adolescents and young adults living in Baltimore, Maryland, elevated neighborhood crime significantly predicted ratings of subthreshold suspiciousness on the SIPS above and beyond other positive symptoms (43). Similarly, Vargas et al. (44) reported that increased neighborhood crime (indexed by publicly available census and FBI data) was significantly associated with increased ratings of subthreshold suspiciousness on the SIPS for individuals at CHR, even when accounting for the effects of neighborhood socioeconomic status.

Findings from the relevant PLE literature yield similar results. Indices of urbanicity, neighborhood deprivation, and neighborhood lead exposure risk were all significantly associated with self-reported PLEs in a large sample of children participating in the ABCD Study (45). Racial and ethnic density has also been significantly associated with PLEs. Among U.S. undergraduate students who identified as racial or ethnic minorities, growing up in a racially-discordant neighborhood (e.g., being Black in a largely Asian neighborhood) was associated with endorsing more self-reported PLEs than individuals who grew up in racially-concordant, racially-mixed, or predominantly White neighborhoods (46). In this same study, individuals reporting a change in their neighborhood ethnic density later in life (after age 12) also endorsed significantly more PLEs than those students who reported no changes. In a large sample of respondents from the Survey of Public-Police Encounters, neighborhood social disconnectedness, defined as the extent to which individuals feel out of place and unwelcome in their own neighborhood, was statistically significantly associated with delusional thinking, including subthreshold delusions of reference and persecution, control, and hallucinations (47). Taken together, these findings highlight the relevancy of a range of neighborhood-related factors in relation to CHR symptoms and assessment.

#### 2.3.1. Clinical considerations

Neighborhood context may impact both clinician and self-reported ratings of psychosis-spectrum symptomatology. Clinicians and researchers alike should make efforts to assess the impact of various neighborhood factors when assessing or treating individuals suspected to be at CHR. This can be accomplished by asking certain follow-up questions during clinician-led interviews like the SIPS (see Table 1 for examples). Failure to assess these factors may contribute to the over-pathologizing of environmentally-adaptive or normative behaviors (e.g., not leaving the house alone and looking over one's shoulder). Alternatively, inaccurate assessment can also lead to under-pathologizing as these neighborhood factors could serve as authentic stressors leading to increased risk of psychosis. Dedicated efforts to avoid both diagnostic errors may help improve the field's largely inaccurate identification of individuals who later develop a psychotic disorder (48).

### 2.4. Trauma

Traumatic events have been heavily and consistently associated with the entire psychosis continuum, ranging from PLEs to psychotic disorders. Recent advances in research regarding trauma and CHR have identified trauma as an integral factor in CHR trajectories. Trauma appears to be more prevalent among individuals at CHR compared to those not at high risk, and those at CHR also report experiencing significantly more subtypes of traumatic experiences compared to control participants (49). A 2015 meta-analysis by Kraan et al. found that childhood trauma, in particular, was highly prevalent, with 86.8% of participants at CHR reporting childhood trauma (50). Another review of trauma in CHR highlighted that individuals at CHR may be at higher risk for physical trauma and endorse history of sexual abuse at higher rates than the general population (51). Within the NAPLS-2 CHR cohort, those who reported experiencing trauma were significantly more likely to also endorse anxiety, depression, and a negative sense of self, with bullying in particular associated with worse functioning (49). This work highlights the important role trauma may play in relation to comorbid symptoms for individuals at CHR. Exposure to traumatic events also appears to be related to positive symptom severity (52). Additionally, a systematic review of the existing literature found that childhood trauma and adversity were significantly associated with higher risk for psychosis, even while accounting for other factors such as genetic risk (53).

Two specific types of potentially traumatic experiences with implications for treatment-seeking and assessment are police encounters and exposure to violence. Though interactions with law enforcement have remained unassessed in the CHR literature, several studies have assessed relations between PLEs and police victimization. In a study conducted with data from the Survey of Police-Public Encounters, a general population survey of adults living in four different cities in the United States, respondents who reported experiences of police victimization were significantly more likely to self-report PLEs, even when accounting for various demographic variables (54). In a follow-up study, paranoid beliefs were significantly and positively associated with expectations of police victimization, but this association was accounted for by past exposures to similar types of victimization (55). Most recently, exposure to police violence (along with several other factors, including a measure of discrimination) was found to statistically explain a substantial portion of the increased risk for PLEs in a national probability sample of young adults (27).

Similarly, exposure to violence has not been explored in North American CHR studies but has been assessed in the context of PLEs. In a large, diverse sample of adult respondents from the Survey of Police-Public Encounters, exposure to intimate partner violence was statistically significantly associated with total PLE endorsement, and the endorsement of specific types of PLEs (i.e., delusional mood, delusions of reference/persecution, delusion of control, and hallucinations) such that the odds of endorsing any of these PLEs significantly increased as scores on self-reported intimate partner violence and childhood adverse experiences increased (47). In another study of the association between community violence exposure (observation or direct experiences of physical harm or threats of physical harm) and PLEs, community violence was found to be a significant predictor of PRIME Screen item scores for White participants only but was non-significant for Black participants, suggesting that these experiences are differentially predictive of PLEs across racialized identities (33). Together, these findings suggest that exposure to violence impacts the likelihood of reporting PLEs in the general population. Exposure to violence may additionally impact clinician-led diagnoses of psychosis-risk status, particularly for assessments of persecutory delusions and perceptual abnormalities (e.g., hearing gunshots when there are none). More research is needed to address how exposure to violence may impact these diagnoses. Finally, it is important to note that not all exposure to violence and police encounters leads to trauma, though their associations with PLEs may be mediated by trauma or stress. Regardless, these experiences have been repeatedly linked with PLEs and the psychosis spectrum, representing significant stressors that may play a role in CHR assessment and treatment.

#### 2.4.1. Clinical considerations

Despite the links between trauma and CHR, there is a lack of research examining interventions for trauma in youth at CHR (51). Nonetheless, given the well-documented associations between trauma and CHR symptomatology and the high rates of trauma among those at CHR, it is clear that being prepared to provide trauma-informed care is an essential component of specialty services for individuals at CHR. The experience of trauma can also be associated with symptoms that may appear similar to psychosis-spectrum symptoms (e.g., dissociative experiences), presenting a challenge for differential diagnosis. Thus, it can be valuable to conduct more formal trauma assessments or administer trauma questionnaires alongside CHR assessments to parse out symptoms that may be better attributed to trauma phenomenology, symptoms that are more characteristic of psychosis-risk symptomatology, or symptoms that overlap across trauma and CHR. Ultimately, these distinctions can clarify symptom presentation and clinical conceptualizations, helping to inform treatment.

Due to the lack of literature concerning police victimization, it may be difficult for assessors to distinguish true psychosis-risk symptoms, from trauma-related symptoms in response to an incident involving the police, from adaptive behaviors in response to police victimization. Making this distinction is important to aid in differential diagnosis between trauma and CHR, to avoid over-pathologizing normative reactions to stressful experiences, and to identify false-positive endorsements of psychosis-risk assessment items. Assessors should be cautious about pathologizing warranted fear or mistrust toward law enforcement among communities that have historically experienced violence and maltreatment at the hands of law enforcement and the justice system. Assessors should also take care not to discount the impact of any interactions with police (e.g., a traffic stop), even without a resulting arrest or physical harm, considering the collective trauma that may be present among populations who have experienced disproportionate harm from law enforcement. Partnerships between the mental health field and the justice system can create conversations that educate stakeholders about issues faced and possible solutions.

Assessment of possible trauma-related symptoms caused by one-time or repeated exposure to violence may be helpful for assessors attempting to identify whether psychosis-risk symptoms are present alongside traumatic symptoms, or if trauma-related disorders better explain endorsement of psychosis-risk experiences (i.e., making a differential diagnosis between CHR and trauma-related symptomatology). Appropriate follow-up questions may also help assessors determine whether an endorsed experience meets the essence of the SIPS question (see Table 1). For example, an individual who has directly experienced or witnessed violence within their family may endorse a need to pay close attention to their surroundings to be safe. Appropriate follow-up questions for assessors may include the temporality, severity, and functional impact of this experience. Probing for these factors can reduce bias (i.e., avoiding pathologizing normative reactions) and aid in differential diagnosis (i.e., inaccurately conceptualizing trauma as CHR).

### 2.5. Immigration status

Similar to other socio-environmental factors outlined in this review, migration and immigration status have been consistently linked with the psychosis spectrum, but much of this work comes from research of full threshold and first-episode psychosis. Specifically, multiple meta-analyses and systematic reviews have observed higher incidence rates and elevated risk for psychosis and schizophrenia among migrants compared to their native-born counterparts (56–61). In terms of potential explanatory mechanisms behind the link between migration and psychosis, one Canadian study explored both social stress mechanisms (e.g., victimization, discrimination, and isolation) and biological mechanisms (e.g., elevated dopaminergic function in the brain), though causal inferences were limited by the cross-sectional nature of the study (62). Beyond a handful of Canadian studies, most of these data come from outside of North America. Given the unique and challenging process of migrating to the United States, more research is needed to understand immigrant status and the psychosis spectrum in this geographic context specifically.

To expand upon prior studies that identified migrant status as a risk factor for psychosis, O'Donoghue et al. (63) pooled results from five large CHR studies, including the NAPLS project, to examine migrant status as a potential risk factor from transitioning from the CHR [or ultra high-risk (UHR)] state to full threshold psychosis. Their meta-analysis showed no statistically significant association between migrant status and risk for transition from CHR/UHR to psychosis (63). The authors note that these findings are somewhat counterintuitive but posit that migrants may be less likely to be identified during their high-risk phase of illness, and more likely to enter first-episode psychosis clinics directly. There has been limited research investigating help-seeking and immigration status among those at CHR, though some have hypothesized that longer duration of untreated psychosis among immigrants may decrease the likelihood that individuals at high risk among this population will present to UHR or CHR services (64).

In terms of psychosis-risk symptoms among migrants, one Brazilian study with a small sample (*N* = 42; *n*_*migrants*_ = 5) found higher levels of “thought disturbances” among participants with migration history compared to those without (65). Beyond this study and those examining the link between migration and transition rates from CHR to full-threshold psychosis, there has been limited research on the connections between immigration status and the CHR phase. As immigration has been identified as a robust risk factor for and predictor of psychosis elsewhere in the world (primarily in western Europe), more work is needed to understand the relations between immigration status and the clinical high-risk phase in immigrants to North America, including implications for service utilization and treatment.

#### 2.5.1. Clinical considerations

Similar to other marginalized groups, immigrants may face discrimination and migration-related stress, which should be considered important context during psychosis-risk assessment. For example, psychosis-risk assessors and clinicians may ask clients whether certain symptoms began occurring before or after the client migrated to the country. Follow-up probes can help reduce bias by establishing temporal precedence of symptoms in the context of migration, which can shed light on the etiology of the symptoms and whether they are more indicative of psychosis-risk processes or normative reactions to the stress of immigration.

Jones et al. (35) surveyed providers, including from the United States and Canada, about practices and policies related to structural disadvantage within early intervention in psychosis services. Providers cited barriers and challenges faced by migrants in particular, such as marginalization, lack of cultural awareness among providers, and barriers to service engagement. Researchers and clinicians in CHR settings may benefit from considering these unique challenges when assessing and treating individuals with migration history. Additionally, tools such as the Cultural Formulation Interview (13) can assist clinicians and assessors in understanding the culture from which the client has emigrated to understand better whether CHR symptoms endorsed may be understood as normative within their culture of origin.

### 2.6. Gender identity

Gender identity has also been explored as an influential factor in the etiology and assessment of psychosis risk. It is important to note that CHR studies are disproportionately conducted with male participants, using a binary view of gender and sex that inherently constrains the interpretation and generalizability of findings. Meta-analyses of schizophrenia and CHR studies show that approximately two-thirds of participants identify as male, a proportion that does not seem to align with epidemiological estimates of male-to-female ratios in psychosis (66–68). One possible reason for this gender gap is that women are systematically excluded from study participation due to overdiagnosis of affective psychotic disorders that render them ineligible for psychosis-risk studies (11, 69–71). Women have also been found to have a later age of onset for psychosis than men, with average onset in their early thirties vs. late twenties. As such, narrow age criteria for studies may systematically reduce the number of women included (72). Overall, among studies in North America of individuals at high-risk for psychosis, detection strategies tailored to considerations of gender are generally lacking (73).

Despite an underrepresentation of women in CHR studies, evidence supports gender differences in psychosis-spectrum symptom expression and functioning. Negative symptoms are significantly more common in men than women in samples at clinical high-risk for psychosis (30, 74). There also appears to be a differential impact of functioning on conversion risk for men compared to women. CHR studies from the NAPLS cohort have found impaired social and role functioning in men compared to women at baseline (29, 75), with social functioning deficits particularly predictive of future conversion to psychosis (76). Despite these differential findings for psychotic symptoms and functioning, the NAPLS consortium found no difference in conversion to psychosis based on gender in individuals at CHR after 2.5-year follow-up (75).

Conclusions regarding differential risk for conversion are inconsistent across different operationalizations of psychosis risk, including ultra-high risk (UHR) criteria. Some UHR studies have found no difference in conversion to psychosis between men and women (77, 78), while others have found a greater proportion of men among those who convert (79). The literature would ultimately benefit from additional longitudinal work assessing conversion risk by gender identity in North American samples at elevated risk for psychosis.

As noted, most research on gender differences in psychosis risk is binary and focuses on cisgender individuals. To our knowledge, no research exists on how psychotic experiences develop in transgender and gender-expansive populations. Studies have shown that compared to their cisgender counterparts, transgender youth are at increased risk for severe psychopathology in general (80–83), a trend that has not been thoroughly studied among those at CHR. Transgender youth also face discrimination and substantial barriers to accessing mental health care (84, 85), including discrimination from healthcare providers, ignorance and insensitivity to transgender needs, and stigma (85, 86). These barriers may impact willingness to engage in specialized CHR services and participate in research.

#### 2.6.1. Clinical considerations

As women tend to present with more affective symptoms than men, assessors should take care to parse out the chronology of affective and psychosis-spectrum symptoms to make accurate decisions regarding the nature and severity of symptomatology in women (i.e., differential diagnosis distinguishing between affective/non-affective psychosis and CHR symptoms). Considering that women generally have a later age of onset for psychosis, they may experience subthreshold symptoms of psychosis for an extended period. This may have implications for developmental considerations in treatment planning (adolescence vs. early adulthood), as well as the assessment of distress and functioning during the CHR phase depending on when in the course of their symptom progression women typically engage with CHR services. Conversely, studies have found that men more commonly experience negative symptoms in the CHR phase. Since negative symptoms are associated with poor motivation and emotional responsiveness, assessors should be wary of how resistance to intervention in men is conceptualized and make concerted efforts to build rapport and maintain engagement with assessment and treatment procedures.

Depersonalization and a loss of sense of self are notable psychosis spectrum symptoms (87, 88) that may be relevant but not necessarily pathological for young people grappling with their gender identity (11). For these reasons, assessors should be aware of the unique experiences of the gender-expansive populations they work with and incorporate an understanding of normative identity development to avoid over-pathologizing bias. For example, when assessing unusual thought content, a question like “Do you ever worry that something might be wrong with your body or your health?” may resonate with transgender individuals experiencing gender dysphoria and body dysmorphia. In this scenario, the assessor would need to probe further to differentiate these contextually normative experiences from somatic delusions. Parsing out psychosis symptoms from gender-related experiences is critical to valid assessment. Assessors should also aim to create an inclusive space in all client interactions. This may involve asking clients their preferred pronouns, sharing one's own pronouns, and being mindful of preferred names. Ultimately, CHR researchers and service providers must make an effort to conduct assessments informed by context and respect the experiences of gender-expansive individuals.

### 2.7. Sexual orientation

Although literature on how sexual orientation relates to psychosis risk is limited, especially from the U.S., European studies suggest that sexual minority status is an important social determinant of risk for psychotic experiences (89–91). Findings in the U.S. show that gay and bisexual men are twice as likely as their heterosexual counterparts to meet for a diagnosis of schizophrenia or psychotic illness (92). Further research is needed to understand how sexual orientation relates to the onset and assessment of psychotic experiences in North America, as sociocultural context shapes the perception and experiences of sexual minority populations (93). This research should incorporate data from SIPS assessments to investigate symptom presentations and functioning of sexual minority individuals in the CHR phase.

#### 2.7.1. Clinical considerations

Given that individuals at CHR report greater perceived discrimination due to sexual orientation compared to those not at risk for psychosis (37), assessors should be thoughtful in their approach to working with sexual minority participants. Due to the longstanding history of the pathologization of queerness, researchers should be transparent about the rationale for collecting information on participants' sexuality and personal experiences. Similar to transgender and gender-expansive individuals, many young people grapple with their burgeoning sexual identity. As such, assessors need to be mindful of conflating sexual identity development in a heterosexist society with psychosis-spectrum experiences to avoid over-pathologizing bias. For example, endorsing a loss of sense of self or unusual and excessive guilt are psychosis-spectrum experiences that might warrant careful consideration in sexual minority populations, as they may be more attributable to feelings of internalized stigma or normative identity development rather than true psychosis-risk phenomenology. In differentiating between these experiences, assessors should probe further as to the origin of the experience. In general, assessors should be mindful of making assumptions based on perceived sexual orientation, such as assuming the gender of an individual's partner. These assumptions may be perceived as disrespectful, hinder rapport, and impact the efficacy of CHR assessment in sexual minority populations.

### 2.8. Age

Given high rates of false positive results on self-report psychosis screening tools (94), age has been proposed as a moderating variable that may impact psychosis-risk screening accuracy. Previous work suggests that younger adolescents report psychotic-like experiences at higher rates than older adolescents (95). Some longitudinal findings suggest that psychotic experiences reported in childhood may be less clinically relevant than psychotic experiences starting in adolescence, as childhood psychotic experiences tend to be transitory and associated with lower rates of psychopathology (96–98). It remains unclear, however, to what degree age impacts psychosis-risk clinician-facilitated assessment.

Rakhshan Rouhakhtar et al. (99) suggest that age may moderate the association between self-report assessment of psychotic-like experiences and clinician-diagnosed clinical high-risk states. In their sample of racially diverse, lower-income, help-seeking adolescents and young adults between the ages of 12 and 23 years old, compared to older participants, younger participants endorsed more items on the PRIME Screen and were more likely to have a false positive screen after clinician interview. These findings speak to the need for improvement in the validity of psychosis screening tools, particularly for young adolescents, and the need to consider chronological age when conducting clinician-led interviews of psychosis risk. Ultimately, earlier age of true symptom onset is associated with conversion to psychosis among individuals at CHR, highlighting the importance of screening accuracy across developmental stages (77).

#### 2.8.1. Clinical considerations

When considering age as a contextual factor that may impact the accuracy of psychosis-risk assessments, accurate diagnosis of true subthreshold psychotic symptoms depends on clinician-generated follow-up questions. This additional probing can ensure that (1) younger respondents, in particular, and the interviewer have a shared understanding of the question at hand, and (2) the interviewer is appropriately differentiating true psychotic experiences (e.g., delusion of mind reading accompanied by some level of distress and impairment) from developmentally normative experiences (e.g., feeling so close with their best friend that they feel as if they know what the other is thinking or feeling). Failure to consider age and developmental context may lead to systemic bias in CHR assessment whereby developmentally normative or transient experiences may be miscategorized as psychosis risk. Additional research is needed to develop guidelines for asking follow-up questions during clinician-led psychosis-risk interviews across varied age groups to promote developmentally sensitive CHR assessment.

## 3. Discussion and call to action

There has been an exponential increase in the amount of literature focusing on the clinical high-risk phase of psychosis. Yet, the field's accuracy in predicting full-threshold psychosis and improving functional outcomes among individuals at CHR has seemingly begun to level. Recent meta-analytic estimates of conversion to psychosis among individuals at CHR suggest that only 22% of identified individuals convert to a full-threshold psychotic disorder within 3 years (18). Additionally, existing early intervention programs largely report non-significant differences in functional outcomes among individuals in specialized early intervention for CHR (7). Given the numerous ethical concerns about assigning a CHR diagnosis to youth (100–102), improving the field's diagnostic accuracy is of the utmost importance.

As outlined by Anglin et al. (9), the past two decades of CHR identification and prevention work have focused on identifying individual-level factors (e.g., neuroimaging and symptom evaluation) that precede the onset of psychotic disorders. Additional research in sociocultural factors may be required to break through the current plateaus in identification and treatment. Incorporating sensitivity to the factors outlined in this review may represent a step toward improving differential diagnosis and reducing systemic bias in CHR screening and assessment, ultimately improving accuracy of CHR identification and quality of treatment.

This gap between research on contextual factors and improved identification of young people at CHR may be in part due to the lack of integration between areas of research that (1) assess aspects of marginalized identities as outlined above, (2) assess these identities in the context of intersectionality, as these sociocultural factors rarely occur in a vacuum, (3) explore the impact of these sociocultural factors on psychosis risk and functional outcomes longitudinally, and (4) test how systematic appraisals of these contextual factors can be integrated into formalized, clinician-led diagnostic interviews of psychosis risk to minimize bias and facilitate accurate differential diagnosis. Recent large-scale, multi-site, longitudinal studies of psychosis risk, such as the Multi-site Assessment of Psychosis-risk Study (MAP Study) (103), seek to address this gap by combining and evaluating traditional psychosis-risk screening tools such as the PRIME Screen and PQ with self-report screenings of contextual factors such as trauma, immigration status, exposure to violence, and other factors explored in this review. Another newly initiated project, the Psychosis-Risk Outcomes Network (ProNET) (104), is a multi-site, international study seeking to better understand the heterogeneity of psychosis-risk trajectories, measuring CHR outcomes such as biomarkers and cognition alongside socio-environmental and contextual factors.

In clinical settings, incorporating context-sensitive practices and policies can help to support clients optimally. Gold-standard tools for identifying psychosis risk, such as the SIPS, can be augmented to be more inclusive of cultural and contextual considerations. For instance, adding standardized questions to the SIPS regarding culture and subculture could provide assessors needed context when evaluating if item endorsement meets the essence of the question as intended by the assessor. In the absence of a SIPS revision, the SIPS and any other measure of psychosis risk could be complemented with tools such as the Cultural Formulation Interview (13) to provide additional contextual information. The field should also be open to the possibility that existing measures may lack cross-cultural validity for some groups of people. In such cases, the creation of new measures that are more culturally responsive should be considered. In addition to the measures themselves, training in their use needs to emphasize cultural responsivity and humility, offering tangible steps clinicians can take to be more contextually informed in their formulation.

In terms of promising intervention practices, early psychosis service providers have noted that standard care-coordination practices, such as connecting clients to social welfare supports, can help with challenges facing marginalized groups but can also be insufficient (35). Tailored strategies such as designing services to address the unique needs of individual clients with the understanding that these individuals may be part of minoritized groups, colocating with family services, and consulting and engaging with cultural leaders and communities can be steps toward contextually responsive CHR services (35, 105). Simply put, it is clear that there is much to be learned as a field in this regard. Listening and co-creating with our clients may be required for us to develop a truly inclusive approach to assessment and treatment.

Although not the focus of this review, we would be remiss if we failed to acknowledge that all of the above considerations are made within a system of care that has historically (further) marginalized minoritized groups. Mistrust of the health and mental health care systems is rooted in practices and policies that began as early as when Europeans colonized North America. More recent decades, including the present, are no exception to the pattern. For example, mental health diagnostic labels related to psychosis are used to oppress voices or inappropriately ascribe responsibility for injustices. Researchers and providers should recognize that, regardless of their intentions, they may be perceived as part of a system of oppression that could contribute to psychosis risk for large swaths of minoritized communities. The field may need to do more in the present to address injustices from the past.

The factors discussed in this review make the assessment and diagnosis of psychosis risk quite nuanced. Failure to take context into account in CHR assessment and treatment services could lead to making clinical interpretive mistakes (e.g., from clinician bias and incorrect differential diagnosis) that could have harmful consequences (100). Integrating different silos of research related to social determinants of CHR and exploring how social, environmental, and contextual factors interrelate and interact with individual-level factors to influence CHR trajectories can help to promote better identification, assessment, and treatment of psychosis risk.

## 4. Conclusions and future directions

The findings outlined in this review suggest that while the field has made considerable progress over the last two decades, certain sociocultural factors have not been adequately addressed in the context of the clinical high-risk phase for psychosis. Sociocultural factors that would benefit from additional research in U.S. contexts include both those included in this review (e.g., immigration, gender identity, sexual orientation, exposure to police violence, neighborhood context, and more) and those not included in this review (e.g., religion/spirituality, societal stigma, cultural beliefs). These knowledge gaps may contribute to the need for standardized guidelines or modifications being proposed and implemented for commonly used psychosis-risk symptom measures, such as the SIPS. Future directions for this niche area include conducting more research on the impact of sociocultural factors on CHR identification, intervention, and functional outcomes using large, nationally representative samples, addressing sociocultural factors that have largely gone unaddressed in North America (e.g., immigration, religion, and spirituality), and translating these findings such that they can be systematically implemented into clinical research and practice.

## Author contributions

MB and EP designed and conducted the review with guidance from JS. MB, EP, and MG wrote the first draft of the manuscript. All authors contributed to the design of the review, edited drafts of the manuscript, contributed to the article, and approved the submitted version.

## References

[1] NIMH. Schizophrenia. Schizophrenia. Available online at: https://www.nimh.nih.gov/health/statistics/schizophrenia (accessed February 12, 2023).

[2] van Os J Linscott RJ Myin-Germeys I Delespaul P Krabbendam L. A systematic review and meta-analysis of the psychosis continuum: evidence for a psychosis proneness–persistence–impairment model of psychotic disorder. Psychol Med. (2009) 39:179–95. 10.1017/S0033291708003814 18606047

[3] van Os J Linscott RJ. Introduction: the extended psychosis phenotype–relationship with schizophrenia and with ultrahigh risk status for psychosis. Schizophr Bull. (2012) 38:227–30. 10.1093/schbul/sbr188 22355185 PMC3283160

[4] McGlashan TH Walsh BC Woods SW. The Psychosis Risk Syndrome: Handbook for Diagnosis and Follow-up. Oxford: Oxford University Press (2010).

[5] Miller TJ McGlashan TH Rosen JL Cadenhead K Ventura J McFarlane W. Prodromal assessment with the structured interview for prodromal syndromes and the scale of prodromal symptoms: predictive validity, interrater reliability, and training to reliability. Schizophr Bull. (2003) 29:703–15. 10.1093/oxfordjournals.schbul.a007040 14989408

[6] Addington J Devoe DJ Santesteban-Echarri O. Multidisciplinary treatment for individuals at clinical high risk of developing psychosis. Curr Treat Options Psychiatry. (2019) 6:1–16. 10.1007/s40501-019-0164-6 31403023 PMC6688178

[7] Mei C van der Gaag M Nelson B Smit F Yuen HP Berger M. Preventive interventions for individuals at ultra high risk for psychosis: an updated and extended meta-analysis. Clin Psychol Rev. (2021) 86:102005. 10.1016/j.cpr.2021.102005 33810885

[8] Okuzawa N Kline E Fuertes J Negi S Reeves G Himelhoch S. Psychotherapy for adolescents and young adults at high risk for psychosis: a systematic review: psychotherapy for high-risk youth. Early Interv Psychiatry. (2014) 8:307–22. 10.1111/eip.12129 24576077

[9] Anglin DM Galea S Bachman P. Going upstream to advance psychosis prevention and improve public health. JAMA Psychiatry. (2020) 77:665. 10.1001/jamapsychiatry.2020.0142 32236511

[10] Anglin DM Ereshefsky S Klaunig MJ Bridgwater MA Niendam TA Ellman LM. From womb to neighborhood: a racial analysis of social determinants of psychosis in the United States. Am J Psychiatry. (2021) 178:599–610. 10.1176/appi.ajp.2020.20071091 33934608 PMC8655820

[11] DeLuca JS Novacek DM Adery LH Herrera SN Landa Y Corcoran CM. Equity in mental health services for youth at clinical high risk for psychosis: considering marginalized identities and stressors. Evid-Based Pract Child Adolesc Ment Health. (2022) 7:176–97. 10.1080/23794925.2022.2042874 35815004 PMC9258423

[12] Schiffman J Ellman LM Mittal VA. Individual differences and psychosis-risk screening: practical suggestions to improve the scope and quality of early identification. Front Psychiatry. (2019) 10:6. 10.3389/fpsyt.2019.00006 30837898 PMC6382740

[13] Lewis-Fernández R Aggarwal NK Kirmayer LJ. The cultural formulation interview: progress to date and future directions. Transcult Psychiatry. (2020) 57:487–96. 10.1177/1363461520938273 32838656

[14] Janssen I Hanssen M Bak M Bijl RV De Graaf R Vollebergh W. Discrimination and delusional ideation. Br J Psychiatry. (2003) 182:71–6. 10.1192/bjp.182.1.71 12509322

[15] Bogart LM Landrine H Galvan FH Wagner GJ Klein DJ. Perceived discrimination and physical health among HIV-positive black and latino men who have sex with men. AIDS Behav. (2013) 17:1431–41. 10.1007/s10461-012-0397-5 23297084 PMC3631464

[16] Krieger N Smith K Naishadham D Hartman C Barbeau EM. Experiences of discrimination: validity and reliability of a self-report measure for population health research on racism and health. Soc Sci Med. (2005) 61:1576–96. 10.1016/j.socscimed.2005.03.006 16005789

[17] First MB Williams JBW Karg RS Spitzer RL. Structured Clinical Interview for DSM-5 Disorders, Clinician Version (SCID-5-CV). Arlington, VA: American Psychiatric Association (2016).

[18] Fusar-Poli P Salazar de Pablo G Correll CU Meyer-Lindenberg A Millan MJ Borgwardt S. Prevention of psychosis: advances in detection, prognosis, and intervention. JAMA Psychiatry. (2020) 77:755. 10.1001/jamapsychiatry.2019.4779 32159746

[19] Petti E Klaunig MJ Smith ME Bridgwater MA Roemer C Andorko ND. Mental health care utilization in individuals with high levels of psychosis-like experiences: associations with race and potentially traumatic events. Cultur Divers Ethnic Minor Psychol. (2021). 10.1037/cdp0000500 34807672

[20] Smith ME Pahwa R Harrison GD Sharpe TL. A social–ecological model for navigating safety across time: the experience of black adults with serious mental illnesses. J Soc Soc Work Res. (2022) 13:353–79. 10.1086/714634

[21] Anglin DM. Racial and ethnic effects on psychotic psychiatric diagnostic changes from admission to discharge: a retrospective chart review. J Clin Psychiatry. (2008) 6:1. 10.1037/e704912007-001 18312062

[22] Minsky S Vega W Miskimen T Gara M Escobar J. Diagnostic patterns in Latino, African American, and European American psychiatric patients. Arch Gen Psychiatry. (2003) 60:637. 10.1001/archpsyc.60.6.637 12796227

[23] Schwartz RC Blankenship DM. Racial disparities in psychotic disorder diagnosis: a review of empirical literature. World J Psychiatry. (2014) 4:133. 10.5498/wjp.v4.i4.133 25540728 PMC4274585

[24] Barrio C Yamada AM Atuel H Hough RL Yee S Berthot B. A tri-ethnic examination of symptom expression on the positive and negative syndrome scale in schizophrenia spectrum disorders. Schizophr Res. (2003) 60:259–69. 10.1016/S0920-9964(02)00223-2 12591588

[25] Eack SM Bahorik AL Newhill CE Neighbors HW Davis LE. Interviewer-perceived honesty as a mediator of racial disparities in the diagnosis of schizophrenia. Psychiatr Serv. (2012) 63:875–80. 10.1176/appi.ps.201100388 22751938 PMC3718294

[26] Karcher NR Klaunig MJ Elsayed NM Taylor RL Jay SY Schiffman J. Understanding associations between race/ethnicity, experiences of discrimination, and psychotic-like experiences in middle childhood. J Am Acad Child Adolesc Psychiatry. (2022) 61:1262–72. 10.1016/j.jaac.2022.03.025 35378237 PMC9525459

[27] DeVylder J Anglin D Munson MR Nishida A Oh H Marsh J. Ethnoracial variation in risk for psychotic experiences. Schizophr Bull. (2022) sbac171. 10.1093/schbul/sbac171 36398917 PMC10016402

[28] Thompson JL Kelly M Kimhy D Harkavy-Friedman JM Khan S Messinger JW. Childhood trauma and prodromal symptoms among individuals at clinical high risk for psychosis. Schizophr Res. (2009) 108:176–81. 10.1016/j.schres.2008.12.005 19174322 PMC2699667

[29] Salokangas RKR Nieman DH Heinimaa M Svirskis T Luutonen S From T. Psychosocial outcome in patients at clinical high risk of psychosis: a prospective follow-up. Soc Psychiatry Psychiatr Epidemiol. (2013) 48:303–11. 10.1007/s00127-012-0545-2 22797132

[30] Corcoran CM Kimhy D Parrilla-Escobar MA Cressman VL Stanford AD Thompson J. The relationship of social function to depressive and negative symptoms in individuals at clinical high risk for psychosis. Psychol Med. (2011) 41:251–61. 10.1017/S0033291710000802 20444306 PMC3376746

[31] Millman ZB Rakhshan Rouhakhtar PJ DeVylder JE Smith ME Phalen PL Woods SW. Evidence for differential predictive performance of the prime screen between black and white help-seeking youths. Psychiatr Serv. (2019) 70:907–14. 10.1176/appi.ps.201800536 31310187 PMC6773467

[32] Miller TJ Cicchetti D Markovich PJ Woods S. The SIPS Screen: a brief self-report screen to detect the schizophrenia prodrome. Schizophr Res. (2004) 70.

[33] Rakhshan Rouhakhtar PJ Pitts SC Schiffman J. Associations between race, discrimination, community violence, traumatic life events, and psychosis-like experiences in a sample of college students. J Clin Med. (2019) 8:1573. 10.3390/jcm8101573 31581531 PMC6832877

[34] López SR. Patient variable biases in clinical judgment: conceptual overview and methodological considerations. Psychol Bull. (1989) 106:184–203. 10.1037/0033-2909.106.2.184 2678201

[35] Jones N Kamens S Oluwoye O Mascayano F Perry C Manseau M. Structural disadvantage and culture, race, and ethnicity in early psychosis services: international provider survey. Psychiatr Serv. (2021) 72:254–63. 10.1176/appi.ps.202000211 33430649 PMC9119303

[36] Gamble VN. Under the shadow of Tuskegee: African Americans and health care. Am J Public Health. (1997) 87:6. 10.2105/AJPH.87.11.1773 9366634 PMC1381160

[37] Saleem MM Stowkowy J Cadenhead KS Cannon TD Cornblatt BA McGlashan TH. Perceived discrimination in those at clinical high risk for psychosis: perceived discrimination in clinical high risk. Early Interv Psychiatry. (2014) 8:77–81. 10.1111/eip.12058 23773288 PMC3883915

[38] Stowkowy J Liu L Cadenhead KS Cannon TD Cornblatt BA McGlashan TH. Early traumatic experiences, perceived discrimination and conversion to psychosis in those at clinical high risk for psychosis. Soc Psychiatry Psychiatr Epidemiol. (2016) 51:497–503. 10.1007/s00127-016-1182-y 26851943 PMC7012367

[39] Anglin DM Lighty Q Greenspoon M Ellman LM. Racial discrimination is associated with distressing subthreshold positive psychotic symptoms among US urban ethnic minority young adults. Soc Psychiatry Psychiatr Epidemiol. (2014) 49:1545–55. 10.1007/s00127-014-0870-8 24695907

[40] Anglin DM Lui F Espinosa A Tikhonov A Ellman L. Ethnic identity, racial discrimination and attenuated psychotic symptoms in an urban population of emerging adults: ethnic identity and APPS. Early Interv Psychiatry. (2018) 12:380–90. 10.1111/eip.12314 26818635

[41] Oh H Cogburn CD Anglin D Lukens E DeVylder J. Major discriminatory events and risk for psychotic experiences among Black Americans. Am J Orthopsychiatry. (2016) 86:277–85. 10.1037/ort0000158 26963179

[42] van Os J Kenis G Rutten BPF. The environment and schizophrenia. Nature. (2010) 468:203–12. 10.1038/nature09563 21068828

[43] Wilson C Smith ME Thompson E Demro C Kline E Bussell K. Context matters: the impact of neighborhood crime and paranoid symptoms on psychosis risk assessment. Schizophr Res. (2016) 171:56–61. 10.1016/j.schres.2016.01.007 26777883

[44] Vargas T Rakhshan Rouhakhtar PJ Schiffman J Zou DS Rydland KJ Mittal VA. Neighborhood crime, socioeconomic status, and suspiciousness in adolescents and young adults at Clinical High Risk (CHR) for psychosis. Schizophr Res. (2020) 215:74–80. 10.1016/j.schres.2019.11.024 31759810 PMC7036021

[45] Karcher NR Schiffman J Barch DM. Environmental risk factors and psychotic-like experiences in children aged 9–10. J Am Acad Child Adolesc Psychiatry. (2021) 60:490–500. 10.1016/j.jaac.2020.07.003 32682894 PMC7895444

[46] Anglin DM Lui F Schneider M Ellman LM. Changes in perceived neighborhood ethnic density among racial and ethnic minorities over time and psychotic-like experiences. Schizophr Res. (2020) 216:330–8. 10.1016/j.schres.2019.11.034 31822432 PMC7374710

[47] Marsh JJ Narita Z Zhai F Fedina L Schiffman J DeVylder J. Violence exposure, psychotic experiences, and social disconnection in an urban community sample. Psychosis. (2022) 14:57–69. 10.1080/17522439.2021.1907774

[48] Fusar-Poli P Cappucciati M Rutigliano G Schultze-Lutter F Bonoldi I Borgwardt S. At risk or not at risk? A meta-analysis of the prognostic accuracy of psychometric interviews for psychosis prediction. World Psychiatry. (2015) 14:322–32. 10.1002/wps.20250 26407788 PMC4592655

[49] Addington J Stowkowy J Cadenhead KS Cornblatt BA McGlashan TH Perkins DO. Early traumatic experiences in those at clinical high risk for psychosis: early trauma in those at CHR for psychosis. Early Interv Psychiatry. (2013) 7:300–5. 10.1111/eip.12020 23343384 PMC3754436

[50] Kraan T Velthorst E Smit F de Haan L van der Gaag M. Trauma and recent life events in individuals at ultra high risk for psychosis: review and meta-analysis. Schizophr Res. (2015) 161:143–9. 10.1016/j.schres.2014.11.026 25499046

[51] Mayo D Corey S Kelly LH Yohannes S Youngquist AL Stuart BK. The role of trauma and stressful life events among individuals at clinical high risk for psychosis: a review. Front Psychiatry. (2017) 8:55. 10.3389/fpsyt.2017.00055 28473776 PMC5397482

[52] Kline E Millman ZB Denenny D Wilson C Thompson E Demro C. Trauma and psychosis symptoms in a sample of help-seeking youth. Schizophr Res. (2016) 175:174–9. 10.1016/j.schres.2016.04.006 27107632

[53] Varese F Smeets F Drukker M Lieverse R Lataster T Viechtbauer W. Childhood adversities increase the risk of psychosis: a meta-analysis of patient-control, prospective- and cross-sectional cohort studies. Schizophr Bull. (2012) 38:661–71. 10.1093/schbul/sbs050 22461484 PMC3406538

[54] DeVylder JE Cogburn C Oh HY Anglin D Smith ME Sharpe T. Psychotic experiences in the context of police victimization: data from the survey of police–public encounters. Schizophr Bull. (2017) 43:993–1001. 10.1093/schbul/sbx038 28369639 PMC5581896

[55] Jun HJ Nam B Fedina L Smith ME Schiffman J Link B. Paranoid beliefs and realistic expectations of victimization: data from the survey of police-public encounters. Schizophr Res. (2018) 199:326–32. 10.1016/j.schres.2018.02.046 29525461

[56] Bourque F van der Ven E Malla A. A meta-analysis of the risk for psychotic disorders among first- and second-generation immigrants. Psychol Med. (2011) 41:897–910. 10.1017/S0033291710001406 20663257

[57] Cantor-Graae E Selten JP. Schizophrenia and migration: a meta-analysis and review. Am J Psychiatry. (2005) 162:12–24. 10.1176/appi.ajp.162.1.12 15625195

[58] Kirkbride JB Errazuriz A Croudace TJ Morgan C Jackson D Boydell J. Incidence of schizophrenia and other psychoses in England, 1950–2009: a systematic review and meta-analyses. Scott JG, editor. PLoS ONE. (2012) 7:e31660. 10.1371/journal.pone.0031660 22457710 PMC3310436

[59] McGrath J Saha S Welham J El Saadi O MacCauley C Chant D. systematic review of the incidence of schizophrenia: the distribution of rates and the influence of sex, urbanicity, migrant status and methodology. BMC Med. (2004) 2:13. 10.1186/1741-7015-2-13 15115547 PMC421751

[60] Radua J Ramella-Cravaro V Ioannidis JPA Reichenberg A Phiphopthatsanee N Amir T. What causes psychosis? An umbrella review of risk and protective factors. World Psychiatry. (2018) 17:49–66. 10.1002/wps.20490 29352556 PMC5775150

[61] Selten JP van der Ven E Termorshuizen F. Migration and psychosis: a meta-analysis of incidence studies. Psychol Med. (2020) 50:303–13. 10.1017/S0033291719000035 30722795 PMC7083571

[62] Egerton A Howes OD Houle S McKenzie K Valmaggia LR Bagby MR. Elevated striatal dopamine function in immigrants and their children: a risk mechanism for psychosis. Schizophr Bull. (2017) 43:sbw181. 10.1093/schbul/sbw181 28057720 PMC5605255

[63] O'Donoghue B Geros H Sizer H Addington J Amminger GP Beaden CE. The association between migrant status and transition in an ultra-high risk for psychosis population. Soc Psychiatry Psychiatr Epidemiol. (2021) 56:943–52. 10.1007/s00127-020-02012-6 33399885

[64] Geros H Sizer H Mifsud N Reynolds S Kim DJ Eaton S. Migrant status and identification as ultra-high risk for psychosis and transitioning to a psychotic disorder. Acta Psychiatr Scand. (2020) 141:52–9. 10.1111/acps.13099 31520527

[65] Nogueira AS Andrade JC Serpa MH Alves TM Freitas EL Hortêncio L. Influence of migration on the thought process of individuals at ultra-high risk for psychosis. Braz J Psychiatry. (2021) 43:285–8. 10.1590/1516-4446-2019-0685 32756811 PMC8136387

[66] Longenecker J Genderson J Dickinson D Malley J Elvevåg B Weinberger DR. et al. Where have all the women gone?: Participant gender in epidemiological and non-epidemiological research of schizophrenia. Schizophr Res. (2010) 119:240–5. 10.1016/j.schres.2010.03.023 20399612 PMC2881627

[67] Rietschel L Lambert M Karow A Zink M Müller H Heinz A. Clinical high risk for psychosis: gender differences in symptoms and social functioning. Early Interv Psychiatry. (2017) 11:306–13. 10.1111/eip.12240 25808791

[68] Tor J Dolz M Sintes A Muñoz D Pardo M de la Serna E. Clinical high risk for psychosis in children and adolescents: a systematic review. Eur Child Adolesc Psychiatry. (2018) 27:683–700. 10.1007/s00787-017-1046-3 28914382

[69] Blazer DG Kessler RC McGonagle KA Swartz MS. The prevalence and distribution of major depression in a national community sample: the National Comorbidity Survey. Am J Psychiatry. (1994) 151:979–86. 10.1176/ajp.151.7.979 8010383

[70] Heitz U Studerus E Menghini-Müller S Papmeyer M Egloff L Ittig S. Gender differences in first self-perceived signs and symptoms in patients with an at-risk mental state and first-episode psychosis. Early Interv Psychiatry. (2019) 13:582–8. 10.1111/eip.12528 29235240

[71] Ochoa S Usall J Cobo J Labad X Kulkarni J. Gender differences in schizophrenia and first-episode psychosis: a comprehensive literature review. Schizophr Res Treat. (2012) 2012:1–9. 10.1155/2012/916198 22966451 PMC3420456

[72] Ferrara M Srihari VH. Early intervention for psychosis in the United States: tailoring services to improve care for women. Psychiatr Serv. (2021) 72:5–6. 10.1176/appi.ps.202000205 32966169 PMC11971724

[73] Barajas A Ochoa S Obiols JE Lalucat-Jo L. Gender differences in individuals at high-risk of psychosis: a comprehensive literature review. Sci World J. (2015) 2015:e430735. 10.1155/2015/430735 25685840 PMC4312997

[74] Willhite RK Niendam TA Bearden CE Zinberg J O'Brien MP Cannon TD. Gender differences in symptoms, functioning and social support in patients at ultra-high risk for developing a psychotic disorder. Schizophr Res. (2008) 104:237–45. 10.1016/j.schres.2008.05.019 18573639 PMC2612126

[75] Walder DJ Holtzman CW Addington J Cadenhead K Tsuang M Cornblatt B. Sexual dimorphisms and prediction of conversion in the NAPLS psychosis prodrome. Schizophr Res. (2013) 144:43–50. 10.1016/j.schres.2012.11.039 23340377 PMC4045468

[76] Tarbox SI Addington J Cadenhead KS Cannon TD Cornblatt BA Perkins DO. Premorbid functional development and conversion to psychosis in clinical high-risk youths. Dev Psychopathol. (2013) 25:1171–86. 10.1017/S0954579413000448 24229556 PMC4356489

[77] Amminger GP Leicester S Yung AR Phillips LJ Berger GE Francey SM. Early-onset of symptoms predicts conversion to non-affective psychosis in ultra-high risk individuals. Schizophr Res. (2006) 84:67–76. 10.1016/j.schres.2006.02.018 16677803

[78] Lemos-Giráldez S Vallina-Fernández O Fernández-Iglesias P Vallejo-Seco G Fonseca-Pedrero E Paíno-Piñeiro M. Symptomatic and functional outcome in youth at ultra-high risk for psychosis: a longitudinal study. Schizophr Res. (2009) 115:121–9. 10.1016/j.schres.2009.09.011 19786339

[79] Ziermans TB Schothorst PF Sprong M van Engeland H. Transition and remission in adolescents at ultra-high risk for psychosis. Schizophr Res. (2011) 126:58–64. 10.1016/j.schres.2010.10.022 21095104

[80] Connolly MD Zervos MJ Barone CJ Johnson CC Joseph CLM. The mental health of transgender youth: advances in understanding. J Adolesc Health Off Publ Soc Adolesc Med. (2016) 59:489–95. 10.1016/j.jadohealth.2016.06.012 27544457

[81] Day JK Fish JN Perez-Brumer A Hatzenbuehler ML Russell ST. Transgender youth substance use disparities: results from a population-based sample. J Adolesc Health. (2017) 61:729–35. 10.1016/j.jadohealth.2017.06.024 28942238 PMC6802742

[82] McClain Z Peebles R. Body image and eating disorders among lesbian, gay, bisexual, and transgender youth. Pediatr Clin North Am. (2016) 63:1079–90. 10.1016/j.pcl.2016.07.008 27865334 PMC6402566

[83] Reisner SL Pardo ST Gamarel KE Hughto JMW Pardee DJ Keo-Meier CL. Substance use to cope with stigma in healthcare among us female-to-male trans masculine adults. LGBT Health. (2015) 2:324–32. 10.1089/lgbt.2015.0001 26788773 PMC4808281

[84] Maguen S Shipherd JC Harris HN. Providing culturally sensitive care for transgender patients. Cogn Behav Pract. (2005) 12:479–90. 10.1016/S1077-7229(05)80075-6

[85] Shipherd JC Green KE Abramovitz S. Transgender clients: identifying and minimizing barriers to mental health treatment. J Gay Lesbian Ment Health. (2010) 14:94–108. 10.1080/19359701003622875

[86] Sperber J Landers S Lawrence S. Access to health care for transgendered persons: results of a needs assessment in Boston. Int J Transgenderism. (2005) 8:75–91. 10.1300/J485v08n02_08

[87] Ferri F Costantini M Salone A Di Iorio G Martinotti G Chiarelli A. Upcoming tactile events and body ownership in schizophrenia. Schizophr Res. (2014) 152:51–7. 10.1016/j.schres.2013.06.026 23835002

[88] Nelson B Fornito A Harrison BJ Yücel M Sass LA Yung AR. A disturbed sense of self in the psychosis prodrome: linking phenomenology and neurobiology. Neurosci Biobehav Rev. (2009) 33:807–17. 10.1016/j.neubiorev.2009.01.002 19428493

[89] Chakraborty A McManus S Brugha TS Bebbington P King M. Mental health of the non-heterosexual population of England. Br J Psychiatry. (2011) 198:143–8. 10.1192/bjp.bp.110.082271 21282785

[90] Jacob L Smith L McDermott D Haro JM Stickley A Koyanagi A. Relationship between sexual orientation and psychotic experiences in the general population in England. Psychol Med. (2021) 51:138–46. 10.1017/S003329171900309X 31694728

[91] Gevonden MJ Selten JP Myin-Germeys I de Graaf R ten Have M van Dorsselaer S. Sexual minority status and psychotic symptoms: findings from the Netherlands Mental Health Survey and Incidence Studies (NEMESIS). Psychol Med. (2014) 44:421–33. 10.1017/S0033291713000718 23710972

[92] Bolton SL Sareen J. Sexual orientation and its relation to mental disorders and suicide attempts: findings from a nationally representative sample. Can J Psychiatry. (2011) 56:35–43. 10.1177/070674371105600107 21324241

[93] Tskhay KO Rule NO. Sexual orientation across culture and time. In:Safdar S Kosakowska-Berezecka N, editors. Psychology of Gender Through the Lens of Culture: Theories and Applications. Cham: Springer International Publishing (2015). p. 55–73.

[94] van Nierop M van Os J Gunther N Myin-Germeys I de Graaf R ten Have M. Phenotypically continuous with clinical psychosis, discontinuous in need for care: evidence for an extended psychosis phenotype. Schizophr Bull. (2012) 38:231–8. 10.1093/schbul/sbr129 21908795 PMC3283149

[95] Kelleher I Connor D Clarke MC Devlin N Harley M Cannon M. Prevalence of psychotic symptoms in childhood and adolescence: a systematic review and meta-analysis of population-based studies. Psychol Med. (2012) 42:1857–63. 10.1017/S0033291711002960 22225730

[96] Bartels-Velthuis AA Jenner JA van de Willige G van Os J Wiersma D. Prevalence and correlates of auditory vocal hallucinations in middle childhood. Br J Psychiatry. (2010) 196:41–6. 10.1192/bjp.bp.109.065953 20044659

[97] Bartels-Velthuis AA van de Willige G Jenner JA van Os J Wiersma D. Course of auditory vocal hallucinations in childhood: 5-year follow-up study. Br J Psychiatry. (2011) 199:296–302. 10.1192/bjp.bp.110.086918 21708881

[98] Bartels-Velthuis AA Wigman JTW Jenner JA Bruggeman R van Os J. Course of auditory vocal hallucinations in childhood: 11-year follow-up study. Acta Psychiatr Scand. (2016) 134:6–15. 10.1111/acps.12571 27009572

[99] Rakhshan Rouhakhtar PJ Pitts SC Millman ZB Andorko ND Redman S Wilson C. The impact of age on the validity of psychosis-risk screening in a sample of help-seeking youth. Psychiatry Res. (2019) 274:30–5. 10.1016/j.psychres.2019.02.020 30780059 PMC7513881

[100] Millman ZB Schiffman J. False-positives and clinical heterogeneity among youth at clinical high-risk for psychosis - clinical and ethical implications for assessment and treatment. J Ethics Ment Health. (2018) 10:1–19.

[101] Mittal VA Dean DJ Mittal J Saks ER. Ethical, legal, and clinical considerations when disclosing a high-risk syndrome for psychosis: disclosing a high-risk syndrome for psychosis. Bioethics. (2015) 29:543–56. 10.1111/bioe.12155 25689542

[102] Schiffman J Horton LE Landa Y Woods SW. Considerations for providing feedback to patients and families regarding clinical high-risk for psychosis status. Schizophr Res. (2022) 244:55–7. 10.1016/j.schres.2022.01.059 35597133

[103] Ellman LM Schiffman J Mittal VA. Community psychosis risk screening: an instrument development investigation. J Psychiatry Brain Sci. (2020) 5:e200019. 10.20900/jpbs.20200019 32944657 PMC7494215

[104] Woods SW,. ProNET: Psychosis-Risk Outcomes Network. NIH (2020). Available online at: https://reporter.nih.gov/project-details/10093852 (accessed February 12, 2023).

[105] Thompson E Millman ZB Okuzawa N Mittal V DeVylder J Skadberg T. Evidence-based early interventions for individuals at clinical high risk for psychosis: a review of treatment components. J Nerv Ment Dis. (2015) 203:342–51. 10.1097/NMD.0000000000000287 25919384

